# Using Community-Based Participatory Research Principles to Develop More Understandable Recruitment and Informed Consent Documents in Genomic Research

**DOI:** 10.1371/journal.pone.0125466

**Published:** 2015-05-04

**Authors:** Harlyn G. Skinner, Larissa Calancie, Maihan B. Vu, Beverly Garcia, Molly DeMarco, Cam Patterson, Alice Ammerman, Jonathan C. Schisler

**Affiliations:** 1 Department of Nutrition, The University of North Carolina at Chapel Hill, Chapel Hill, North Carolina, United States of America; 2 Center for Health Promotion and Disease Prevention, The University of North Carolina at Chapel Hill, Chapel Hill, North Carolina, United States of America; 3 Presbyterian Hospital/Weill-Cornell Medical Center, New York, New York, United States of America; 4 McAllister Heart Institute, The University of North Carolina at Chapel Hill, Chapel Hill, North Carolina, United States of America; 5 Department of Pharmacology, The University of North Carolina at Chapel Hill, Chapel Hill, North Carolina, United States of America; Geisinger Clinic, UNITED STATES

## Abstract

**Background:**

Heart Healthy Lenoir is a transdisciplinary project aimed at creating long-term, sustainable approaches to reduce cardiovascular disease risk disparities in Lenoir County, North Carolina using a design spanning genomic analysis and clinical intervention. We hypothesized that residents of Lenoir County would be unfamiliar and mistrustful of genomic research, and therefore reluctant to participate; additionally, these feelings would be higher in African-Americans.

**Methodology:**

To test our hypothesis, we conducted qualitative research using community-based participatory research principles to ensure our genomic research strategies addressed the needs, priorities, and concerns of the community. African-American (n = 19) and White (n = 16) adults in Lenoir County participated in four focus groups exploring perceptions about genomics and cardiovascular disease. Demographic surveys were administered and a semi-structured interview guide was used to facilitate discussions. The discussions were digitally recorded, transcribed verbatim, and analyzed in ATLAS.ti.

**Results and Significance:**

From our analysis, key themes emerged: transparent communication, privacy, participation incentives and barriers, knowledge, and the impact of knowing. African-Americans were more concerned about privacy and community impact compared to Whites, however, African-Americans were still eager to participate in our genomic research project. The results from our formative study were used to improve the informed consent and recruitment processes by: 1) reducing misconceptions of genomic studies; and 2) helping to foster participant understanding and trust with the researchers. Our study demonstrates how community-based participatory research principles can be used to gain deeper insight into the community and increase participation in genomic research studies. Due in part to these efforts 80.3% of eligible African-American participants and 86.9% of eligible White participants enrolled in the Heart Healthy Lenoir Genomics study making our overall enrollment 57.8% African-American. Future research will investigate return of genomic results in the Lenoir community.

## Introduction

This paper discusses the Heart Healthy Lenoir (HHL) Genomics Study and the use of community-based participatory research (CBPR) to engage a rural at-risk community in a genomic research study. The HHL Genomics Study is one-third of a larger project designed to create long-term, sustainable approaches to reduce cardiovascular disease (CVD) risk disparities in Lenoir County, North Carolina. The primary aim of the study is to explore the genomic factors associated with CVD risk, clinical outcomes, and responsiveness to CVD risk reduction interventions. Participants were recruited from two clinical interventions, the HHL Lifestyle Study (ClinicalTrials.gov number: NCT01433484) and the HHL Hypertension Control Study (ClinicalTrials.gov number: NCT01425515) [1,2].

Lenoir County, North Carolina was chosen for the HHL study for a variety of factors including its geographical location in eastern North Carolina, high poverty levels, and the community infrastructure. Situated in the heart of the “Stroke Belt”, North Carolina has heart disease, stroke and obesity rates well above the national averages [3,4]; Lenoir County rates are elevated further still over North Carolina’s averages [5]. According to 2014 U.S. Census Bureau estimates, 24.9% of Lenoir County residents lived in poverty between 2008 to 2012 which is 8.1% higher than state averages for the same period [6]. The county is also home to other clinical and public health efforts, including a state-of-the-art hospital, a federally-funded community health center, multiple primary care practices, a local public health department, a revitalized farmers’ market, and a community alliance dedicated to improving the county’s health [7].

To our knowledge, prior to our study there have been no genomic studies performed in Lenoir County. As such, we used CBPR to engage the community and to learn how best to implement our study. Minkler (2010) defines CBPR as “a process that involves community members or recipients of interventions in all phases of the research process” [8]. The CBPR method not only strengthens the relationship between research institutions and their communities, but also increases community ownership of health-promoting programs [9]. In addition, the use of CBPR is an important component of medical research when trying to overcome the mistrust of health researchers by vulnerable groups [10,11]. According to 2013 U.S. Census reports, 40.9% of Lenoir County residents are African-American, compared to 22.0% statewide [6]. African-Americans are considered a vulnerable group due to a history of mistreatment by and lack of consent for medical research conducted on this population (e.g. Henrietta Lacks and the Tuskegee Syphilis Study). Therefore, researchers need to be sensitive to issues of power and historical context when conducting studies on populations that include African-Americans. Corbie-Smith et al. (1999) observed high levels of mistrust regarding medical research amongst African-American focus group (FG) participants [11]. Many in that study mentioned concerns stemming from the Tuskegee Syphilis Study, as well as having a general feeling that they are exploited within the medical research field. The Tuskegee Syphilis Study has a continuing legacy that can impact the relationship between African-Americans and medical research [11–14]. CBPR provides a way for university researchers to hear and address community concerns including any historic misgivings in order to promote program feasibility, acceptability, and success within the community. These methods can be used to develop study materials for the whole community with particular salience for vulnerable groups within the community [15–18]. In particular, improvements to recruitment and informed consent documents can be guided through the use of CBPR methods [19].

In this paper, we present our findings from a CBPR study where we engaged members of a rural community that includes a high proportion of African-Americans. Our objective was to learn how to design study materials that would instill trust and encourage participation in potential research participants, particularly those that are historically under-represented in genomic research using feedback and knowledge derived directly from the community.

## Methods

### Participants

We conducted four FG discussions attended by a total of 35 individuals from Lenoir County that were organized into two African-American (AA) and two White (W) FGs. We used purposeful sampling to ensure that the predominant racial groups in the county, African-American and White, were equally represented in our sample. Our recruitment goal was to have racially homogenous groups with a balance of men and women. Eligibility criteria included being an adult aged 18 and older, English-speaking, and a current resident of Lenoir County. Participants were recruited by key community members (e.g. the Health Director), or through flyers posted in the community. Interested participants were screened by phone to determine eligibility.

### Focus Group Guide Development

Since we were unaware of any previous genomic studies conducted in Lenoir County, we wanted to understand the thoughts, feelings, and concerns both about genomics and heart health from Lenoir residents. Co-investigators with experience in the Lenoir community and community member assistants worked together to develop a semi-structured discussion guide to explore the acceptability of genomic research in Lenoir County based on input from discussions with key community residents. The community member assistants were either referred by our Community Advisory Council or recruited through a job advertisement in the community. The two community member assistants reflected the racial makeup of each FG, either African-American or White. The community residents who helped develop the guide included Lenoir County Health and Human Services agency employees. Based on community input and existing literature, we constructed our guide with the hypothesis that there would be unfamiliarity and mistrust of genomic research in Lenoir County, and reluctance to participate in a genomics study; furthermore, the mistrust and reluctance would be higher in African-Americans.

### Focus Group Protocol

The University of North Carolina Chapel Hill Institutional Review Board reviewed and approved the study protocol (IRB # 10–0395). The FGs were conducted in winter 2011 with each session lasting approximately 90 minutes. Groups were held in a private location at the community hospital. A trained co-investigator with extensive qualitative expertise moderated discussions, assisted by a community member of the research team. At the beginning of each group, the moderator read the consent form aloud and gave participants the opportunity to ask study-related questions before those interested signed the written informed consent form. Next, demographic information was collected via survey (e.g. age, race, and education level). Word association was then used to assess baseline familiarity with the term ‘genomics’. The FG leader then provided an analogy that investigators and community research team members developed to help participants define genomics. Next, participants were asked to verbally rate on a scale from 1 to 10, with 10 being “completely important,” how important genomics is to their health. The discussion then commenced covering the following topics: (1) community concerns about genomics; (2) thoughts and perceptions about genomics and heart health; and (3) community concerns about participation in genomic research. Participants were each paid $25 upon completion of the session.

### Analysis

FGs were digitally recorded and reviewed for quality and completeness. Files were transcribed verbatim then verified by listening to the original recordings. To analyze our data, we first created a coding scheme using both deductive and inductive methods [20,21]. A codebook was developed and applied to organize text and assist with interpretation. Using a deductive *a priori* approach, we developed a codebook based on the discussion topics, the hypothesis, and a preliminary reading of the transcripts before beginning an in-depth analysis of the data. We then incorporated data-driven inductive coding techniques as described by Strauss and Corbin [22], and Crabtree and Miller [23] to explore patterns. We applied the codes from the codebook to each line of transcript text to identify meaningful units of text, connected the codes and identified themes, and confirmed the findings through a process of clustering the themes [23]. While codes were mutually exclusive, lines of text could have been marked with multiple codes if more than one theme was represented.

We used a qualitative data analysis software program, ATLAS.ti 6.2, to facilitate analysis. After each transcript was imported into the software and coded, we retrieved text on specific codes or combination of codes to enable thematic analysis of particular topics [24]. From this, we looked at the quotes in the context of the documents and assessed the levels of agreement and saliency of themes. Finally, we summarized our findings and chose quotes representative of each theme for presentation.

## Results

### Demographics

A total of 35 participants attended the FGs, with 8–10 attendees per group (Table 1). Across all FGs, participant ages ranged from 22 to 86 years with the average age being 57 years. Over half the participants were self-identified African-Americans (n = 19). The majority of participants were females (n = 23). All participants finished high school with approximately half having a college degree or higher. Compared to 2013 U.S. Census Bureau statistics for Lenoir County [6], our FGs had more African-Americans (due to purposeful sampling), more females (66% in the FGs versus 52.2% in Lenoir County), and a higher education level (100% finishing high school and 51% having a Bachelor’s degree or higher in the FGs versus 77.8% and 14.1%, respectively, in Lenoir County). The majority of FG participants also had a known family history of heart disease (n = 23) and had health insurance (n = 24).

**Table 1:** Characteristics of Focus Groups.

Demographics	FG1	FG2	FG3	FG4	All FGs
African-American	100%	100%	—-	—-	54%
	N = 9	N = 10			N = 19
White	—-	—-	100%	100%	46%
			N = 8	N = 8	N = 16
Age (SD)	48 y (10)	57 y (5)	65 y (13)	60 y (20)	57 y (14)
Age Range	22y – 56y	48y – 67y	47y – 85y	38y – 86y	22y – 86y
Gender					
Male	56%	10%	38%	38%	34%
	N = 5	N = 1	N = 3	N = 3	N = 12
Female	44%	90%	62%	62%	66%
	N = 4	N = 9	N = 5	N = 5	N = 23
**Education**					
High School Graduate	100%	100%	100%	100%	100%
	N = 9	N = 10	N = 8	N = 8	N = 35
Bachelor’s Degree or higher	56%	50%	25%	75%	51%
	N = 5	N = 5	N = 2	N = 6	N = 18
**Family History of Heart Disease**					
Yes	33%	70%	75%	88%	66%
	N = 3	N = 7	N = 6	N = 7	N = 23
No	45%	20%	25%	12%	26%
	N = 4	N = 2	N = 2	N = 1	N = 9
Don’t know	22%	10%	—-	—-	8%
	N = 2	N = 1			N = 3
**Health Insurance**					
Yes	44%	70%	62%	100%	69%
	N = 4	N = 7	N = 5	N = 8	N = 24
No	44%	30%	38%	0%	29%
	N = 4	N = 3	N = 3	N = 0	N = 10
Non-response	12%	—-	—-	—-	2%
	N = 1				N = 1

Data are presented as mean (±standard deviation), range, or percent as indicated.

### Defining Genomics

Only one to two people per FG were familiar with the term “genomics”. Soliciting words and ideas participants associated with “genomics” prior to providing the analogy yielded widely varied responses such as *“geometry”*, *“informative”*, and *“a continuing of possibilities”*. The genomics analogy was then given to provide a conceptual framework for participants to better articulate their perspectives during the remainder of the focus group.

*“Genomics is a term that describes the study of all of a person’s genes (their genome)*, *including how genes interact with each other and with the person’s environment*. *This is different from genetics*, *which is the study of a single gene in isolation*. *Think of genomics as a garden and genetics like a plant in your garden*. *If the plant is not flowering*, *you could study just the plant itself (genetics) or look at the surroundings to see if it is too crowded or there is not enough sun (genomics)*.*”*

Following the provision of the analogy, participants verbally reported finding the analogy helpful in understanding what we meant by genomics for our discussion. Participants also made connections between our genomics analogy and family history of common chronic diseases (e.g. CVD, type 2 diabetes, or cancer). Participants’ responses suggest that they understood the distinction between single gene diseases and genomics, which was the purpose of our analogy. Participants then verbally reported genomics as being highly important to their health ranking it an 8.5 out of 10, with 10 being “completely important.” This importance did not vary across racial groups.

### Focus Group Themes

Five themes emerged from the discussion about genomics: transparent communication, privacy, participation incentives and barriers, knowledge, and the impact of knowing. There were no major thematic differences between FGs or racial groups; therefore the results for each theme are representative of all participants regardless of race. Places where one racial group spoke on additional information (e.g. Theme 2: Privacy and Theme 4: Knowledge) are noted within the theme. Below we present a summary of each theme and how we used that information to tailor our materials.

### Theme 1: Transparent Communication

#### Results

Participants found genomics to be a highly *“technical word”* and requested that researchers use simple, non-technical language instead. This theme arose when discussing how to describe the project to the community as well as how to describe the risks and benefits of participation. Participants wanted plain-speak, and specificity regarding research aims and the possibility of receiving negative health information. Specifically, they wanted research staff to be up-front about any risks involved with the study.

*“I would like to know what you’re really looking for*. *Talk to me*.*”* ~ AA

*“Be honest and tell it like it really is*, *and don’t try to sugar coat it*.*”* ~ W

Participants felt that technical language could obfuscate the presence of risk either through poor communication of what would happen to their genomic data or poor communication of what could happen to their health as a result of their “genomics.” Contrary to our hypothesis, there were no major thematic differences between racial groups.

#### Implications

Based on this finding, we incorporated transparent communication into HHL Genomics materials. As such, we were purposeful in using non-technical language in study recruitment and informed consent materials. Our goal was to have transparent communication that fostered trust between researchers and the community. Working with our community team members, we honed language that was easily understood. For example, instead of describing the HHL Genomics study aim as “determining whether genomic signatures can be used to predict responsiveness to interventions that underlie CVD disparities”, the aim was described as “wanting to learn more about genetic factors related to heart disease and needed treatments.”

### Theme 2: Privacy

#### Results

Participants discussed privacy concerns about the handling of personal information and blood. Participants wanted transparent communication about who would have access to their data and that “qualified” professionals would handle blood draws (e.g. research staff, physicians, or nurses). Participants also wanted explicit assurances that their personal information would be protected, particularly regarding how their blood was handled after the study. If these privacy concerns were addressed, the majority of participants stated that they would not have barriers against participation in a genomics study.

*“I would like to know about the privacy part [as an incentive to participate]*.*”* ~ W

*“At the hospital though*, *after they test your blood or use your blood for whatever purpose*, *isn’t that blood destroyed*?*… How do you know that’s what’s happening [here]*? *They show [information leaks] on TV on the Sci-Fi channel and everything*.*”* ~ AA

African-Americans spoke of a mistrust of medical research that Whites did not. More than half of African-Americans voiced mistrust of science in general and of medical professionals even mentioning conspiracy theories. Their main privacy concern was of being identified from their genetic information. Despite this and in contrast to expectations, most African-American participants voiced great trust in HHL researchers and even eagerness to participate in our genomic study.

*“Even though we’re not identifying ourselves and you say after this you know nobody would know anybody*. *How do we know down the road somebody won’t find out who we are*? *And that’s the only inhibitions that I have*.*”* ~ AA

#### Implications

The finding about privacy led to the stressing of de-identification of samples and participant rights under the Genetic Information Non-discrimination Act (GINA) in the informed consent form. Also, recruitment and informed consent materials explicitly discuss the handling of blood samples. While the study did decide to keep samples for future research, it was emphasized that these samples are de-identified and that participants have the right to withdraw their blood from the study at any point in time.

### Theme 3: Participation Incentives and Barriers

#### Results

Receipt of money was named as an incentive for the time and financial costs of study participation (e.g. travel to the study site). Conversely, the concern that participation would require payment for genomic analyses and blood draws was stated as a barrier to participation.

*“[A participation incentive would be that] the services are gratis and medicine can be that way*. *And they cover travel*.*”* ~ W

*“Prevention*. *It’s like…be real positive about prevention*, *heart disease and prevention of heart disease*.*”* ~ AA

Once the FG moderator explained that study participation would be free, participants stressed the importance of explicitly stating that in study materials so that financial concerns would not be a barrier to participation.

*“[Researchers would benefit from] just recognizing that people are different before you go through with the study*. *I would figure out if people do want an incentive with money or people just want to do it just to figure out the information about their genes*.*”* ~ AA

Participants also repeatedly spoke of receiving CVD results from our genomic study. Not only was this an important incentive unto itself, but participants also spoke of using this information to change their lifestyle. Participants wanted incentives that would support lifestyle changes, such as improving diet or increasing physical activity. For example, health center memberships, gym shoes, and support in preparing healthy meals were mentioned. There were no major differences between racial groups for this theme.

#### Implications

From this finding, we learned the importance of stating all monetary gifts and expenses (or lack thereof) upfront and explicitly in our recruitment materials. In addition to the monetary incentive payment schedule, our materials also included explicit statements that participation in our program, which included blood draws and genomic analysis, would not cost participants anything, though transportation to the study site was at the participants’ expense. For example, after explaining the study protocols, the informed consent document stated, “The program is free, but travel costs to and from the measurement visits are not covered.” Additionally, we learned that participants saw value in their genomic CVD results and wanted that information returned to them. Lifestyle supports were strengthened in the two HHL clinical studies as a result of these FGs. HHL Lifestyle participants were provided with healthy recipes and HHL Hypertension Control participants were provided with home blood pressure monitors.

### Theme 4: Knowledge

#### Results

Participants stated interest in genomics for societal, health, and individual benefits.

*“I would love to be part of the solution*.*”* ~ W

Contributing to the larger genomics knowledge pool was named as important by both White and African-American participants. Additionally, African-Americans spoke specifically of adding to the knowledge pool about African-Americans.

*“We need to know these things [results of genomics research] and we need more support in these [African-American] communities*, *they’re not as tight knit as they used to be… we need a lot of this education*.*”* ~ AA

All participants stated interest in the perceived health benefits of genomic knowledge. Particularly, they believed that genomics could yield knowledge about disease states like CVD and Type 2 Diabetes and could then be used to improve their own health and the health of future generations of their family. Again, African-Americans shared this sentiment but were also interested in the perceived health benefits of genomic research for the African-American community at large. Lastly, participants were interested in genomics for the perceived individual benefits. In general, participants viewed personal genomic information as an added catalyst to make lifestyle changes, and the vast majority of participants wanted individualized genomic feedback from the study.

*“If I was to do it [the study] I would want some feedback on my results and also the overall findings on what I could do to change if I had to do any changes*.*”* ~ AA

The desire for returning genomic results was evident irrespective of race. Additional quotes can be found in S1 Table.

#### Implications

This finding led us to explicitly state in our recruitment and informed consent materials that the HHL Genomics study was being performed to help *society*. We did this to avoid ‘therapeutic misconception’ in our materials since our FG participants seemed equally if not more motivated to participate in genomic research due to perceived health benefits or the possibility of receiving genomic results. Appelbaum defines therapeutic misconception as the conflation of research goals with therapeutic treatment [25]. Furthermore, we also stated in our recruitment and informed consent materials that participants would not receive their individual results. Materials stated that since “the results of the blood tests for genomics is not a routine test and would not be easy to understand by either you or your doctor, we will not send you the results of these tests.”

### Theme 5: The Impact of Knowing

#### Results

Value of Knowing: The consensus among the FGs was that the knowledge gained in genomic studies would benefit both society and the individual; furthermore, this information would lead to better health decisions. Some participants expressed negativity about receiving personal genomic information.

*“You have some that want to know the future*, *you have some that don’t want to know the future*. *And I guess [knowledge] can be harmful in some ways too*, *knowing too much*.*”* ~ W

Concerns regarding genomic knowledge revolved around the fear of a “pre-determined” disease state, confusion around what genomic results would mean, and possible depression and stress from thinking about their “genomics” all the time.

Perceived Control: The majority of participants reported feeling that they were ultimately in control of their health no matter what their “genomics” said. Repeatedly, participants alluded to genomic knowledge as something to empower them in making health decisions. Alternatively, others took the perspective that they could not change what was in their genes, sometimes citing family disease history as justification. However, the vast majority of participants, regardless of race, felt that lifestyle choices were controllable (e.g. smoking, diet, and exercise), and that having genomic information would empower them to make lifestyle choices.

*“It’s not really a study but it’s a group that’s helping us to more [or] less take charge of our well-being as far as our health*, *eating right*, *and doing the right things as far as you know keeping our health intact*. *So I would try to participate in as many studies as I have to*, *to take care of* *me*.*”* ~ AA

#### Implications

Our findings suggest that knowing individualized genomic results is highly valuable and empowering to our FG participants, therefore we explicitly addressed this in our recruitment and informed consent materials. Materials explicitly stated that individualized results would not be returned. HHL made this decision due to researchers not anticipating return of genomic results being so coveted in this community and not having genetic counselors in our research plan. Given the range of responses about knowing genomic information, counselors and other forms of support were deemed to be ethically necessary if HHL were to return individualized results to the community.

## Discussion

The goal of HHL is to reduce CVD risk disparities in Lenoir County, NC. In order for our interventions to succeed, we needed a strong relationship with the community as well as the ability to enroll a representative sample of the population into our study. Our approach was to use CBPR principles to tailor the recruitment and informed consent processes to both foster trust and transparency in our relationship with the community and meet our recruitment goals.

Through FGs, we found that participants were not very familiar with the term genomics. This is consistent with previous studies, which demonstrate genetic knowledge to be low nationwide [26,27]. Christianson et al. (2010) replicated this finding in North Carolina and also demonstrated a racial difference in understanding where African-Americans more frequently reported less genomic knowledge [28]. Regardless, this did not seem to diminish our participants’ desire to participate in genomic research. Our hypothesis was that Lenoir County residents would be unfamiliar and mistrustful of genomic research and would therefore be reluctant to participate; we believed this mistrust and reluctance would be higher in African-Americans. All participants voiced trust in HHL researchers and a willingness to participate in our genomic study. While African-Americans did speak of a legacy of mistrust and their privacy concerns stemming from that, they simultaneously voiced trust in HHL researchers and a willingness to participate in our genomic study. Other studies have also reported positive attitudes towards participation in genomic research [29]. However contrary to some published reports [30], our FG participants did not report a difference in willingness to participate by race (enrollment into HHL Genomics by our 35 FG participants was not tracked). Irrespective of race, participants expressed two distinct sentiments about genomic knowledge: knowledge as empowering and knowledge as predetermination. The Protection Motivation Theory could explain these divergent viewpoints. The theory postulates that those with increased perceived threat may engage in protective health behaviors while those who believe that people have no control over their health may lose motivation to engage in protective health behaviors [31]. Research shows that an individual’s threat beliefs can predict their perceived control in response to genomic knowledge [32]. Genomic knowledge was a major participation incentive for our FG participants. The majority of participants wanted the option to obtain individualized genomic results. Returning genomic data has been documented in other several populations [33–36]. In accordance with CBPR principles, investigators explored ways to address community wants while being cognizant of debate in the field as to whether individual results should be returned and how that should be done [37,38]. Ultimately, we determined that HHL did not have the infrastructure to responsibly return results (e.g. genetic counselors) but as a result of the findings presented here, we did initiate an ancillary study investigating methods to return the HHL Genomics Study results to the Lenoir community. Future genomic research studies may consider the question of returning results early in the planning process in order to be responsive to community wants.

Limitations in this study include positive bias towards research since our participants live in an area with other public health interventions and opted to join the FGs, indicating at least some level of trust and interest in medical research. Also, after providing our analogy the participants verbally reported finding the analogy helpful but we did not perform word associations or use any other method to determine if participants’ descriptions of genomics changed after receiving the analogy. Lastly, this data is representative only of the individuals in the FG, which was purposefully sampled to ensure equal representation of the predominant racial groups in the county; all possible opinions of the larger populations may not have been captured. Experienced moderators ensured that all voices were heard in the group to gain the broadest representation of opinions possible. Future studies may benefit by utilizing other data collection methods that allow anonymity, such as a survey, which may elicit divergent views that people may be uncomfortable voicing within a group.

Strengths of this study include that, to our knowledge, this is the first study to include a lay-analogy in defining genomics for participants, which seemed to help frame the conversation. Another strength of this study is the use of CBPR. Community research team members helped develop the FG guide and administer the FGs. Also, our study engaged populations traditionally under-represented in medical research, specifically African-Americans and those from underserved rural areas. Employing CBPR methods of co-learning to build trust between researchers and community members allowed researchers to explore the presence of and remedies to misconceptions and suspicions about medical research within the Lenoir community.

Overall, this study provided valuable information on the motives of potential genomic research participants in Lenoir County as well as ways to use that information to tailor informed consent and recruitment materials. These efforts resulted in not only high participation in our study, but also more African-American than White participants, which is contrary to much of the previous literature [29,30]. The HHL Genomics Study enrolled 253 African-American participants and 185 White participants, representing 82.8% of the eligible participant pool. Of the eligible African-American participants, 80.3% enrolled in our study thereby making the HHL Genomics participant population 58% African-American. We believe that using CBPR methods to elicit the community voice and accordingly adjust study materials and communications yielded a meaningful consent and recruitment process that enabled us to recruit a high percentage of our eligible population and particularly the eligible African-American population. We also believe that CBPR methods are generalizable to other genomic research endeavors and could be used to improve genomic study participation in historically underserved areas as well as in minority populations.

## Supporting Information

S1 Table Additional Quotes about Knowledge. S1 Table provides additional quotes relating to Theme 4.
(DOC)
