# Supplementary material for: Using Community-Based Participatory Research Principles to Develop More Understandable Recruitment and Informed Consent Documents in Genomic Research
Source: PLoS One. 2015 May 4;10(5):e0125466. doi: 10.1371/journal.pone.0125466 (PMC4418607; doi:10.1371/journal.pone.0125466)
Supplement: S1 Table — provides additional quotes relating to Theme 4. (DOC) [file pone.0125466.s001.doc]

**Table S1. Additional Quotes about Knowledge.**

| **Societal Benefits** | - *“If people are willing to contribute in a study that would help somebody, […] as a human of humanity or humane person, I think people should.” (AA)* |
| --- | --- |
|  | - *“[For] the individual impact on the study or individual, and the information that will be provided. And then also the benefits the overall benefits that it’s going to make on the community and society in general the study will impart.” (AA)* |
|  | - *“There needs to be some more research and study on African American community stress and the heart . . . we worry more and it [has] got to do with economics a whole lot of the time . . . the alcohol consumption, the smoking cigarettes, the over-eating [of] comfort foods. Those are our stress relievers” (AA).* |
|  | - *“The overall benefits that it’s going to make on the community and society in general” (AA)* |
|  | - *“I wouldn’t need anything [to participate in a genomics study]. Just to know that you might be helping.” (W)* |
|  | - *“It might prolong somebody else’s [life].” (W)* |
|  | - *“Might save somebody’s life” (W)* |
|  | - *“Wouldn’t need any incentive” (W)* |
| **Health Benefits** | - *“Find out if you have personal disposition to heart disease.” (W)* |
|  | - *“And if you have the knowledge, [you have] accountability for your own self, for your own actions.” (AA)* |
|  | - *“[To] prevent some things from ever happening” (W)* |
|  | - *“I would like to have more information too because just like he said there’s 14 of us [children] . … It’s probably 10 of us is diabetic because Mama and Daddy [are] diabetics. … And if I could do something to stop that. If I just know some more information to help me so my grandbabies won’t get it, I’d like that” (AA)* |
|  | - *“What would motivate me is that if I could do anything to help somebody else.” (AA)* |
|  | - *“if I knew I was susceptible to a certain disease I wouldn’t claim it but I […] would try to learn more about it. I wouldn’t be afraid. I would learn something about it what I do to prevent that.” (AA)* |
| **Individual Benefits** | - *“I definitely would like the report back. […] That would be payment enough.” (W)* |
|  | - *“Like the history. You know where you are coming from and you [are] going to know where you are going.” (AA)* |
|  | - *“I think knowledge is the best incentive.” (AA)* |
|  | - *“So the more I know the better off I am.” (AA)* |
|  | - *“I would like somebody to follow up with me and tell me what I could do to change.” (W)* |
|  | - *“[I’d like to] make the changes [to] live longer […] or comfortabler.” (W)* |
|  | - *“My brother was 36 he died of cancer. And if I had more information to prevent from getting it, I would do it.” (AA)* |

Table S1 provides additional quotes about Theme 4: Knowledge.
